# The coffee diterpene kahweol enhances sensitivity to sorafenib in human renal carcinoma Caki cells through down-regulation of Mcl-1 and c-FLIP expression

**DOI:** 10.18632/oncotarget.20541

**Published:** 2017-08-24

**Authors:** Kyoung-Jin Min, Hee Jung Um, Jee In Kim, Taeg Kyu Kwon

**Affiliations:** ^1^ Department of Immunology, School of Medicine, Keimyung University, Daegu 704-701, South Korea; ^2^ Department of Molecular Medicine, School of Medicine, Keimyung University, Daegu 704-701, South Korea

**Keywords:** sorafenib, kahweol, Mcl-1, c-FLIP

## Abstract

Sorafenib is approved for the treatment of hepatocellular carcinoma (HCC) and advanced renal cell carcinoma (RCC). However, low tumor response and side effects have been widely reported. Therefore, to improve the efficacy of sorafenib, we investigated whether combined treatment with sorafenib and kahweol, the coffee-specific diterpene, has a synergistic effect on apoptotic cell death. Combined treatment with sorafenib and kahweol markedly induced caspase-mediated apoptosis in renal carcinoma Caki cells. Combined treatment with sorafenib and kahweol induced down-regulation of Mcl-1 and c-FLIP expression. We found down-regulation of Mcl-1 and c-FLIP expression was modulated by the ubiquitin-proteasome pathway. Ectopic expression of Mcl-1 inhibited sorafenib plus kahweol-induced apoptosis. Interestingly, combined treatment with sorafenib and kahweol induced apoptotic cell death in c-FLIP overexpressed cells. In addition, combined treatment with sorafenib and kahweol markedly induced apoptosis in human lung carcinoma (A549) and breast carcinoma (MDA-MB-361) cells, but not in human normal mesangial cells and human skin fibroblast cells (HSF). Collectively, our study demonstrates that combined treatment with sorafenib and kahweol induces apoptotic cell death through down-regulation of Mcl-1 expression.

## INTRODUCTION

Sorafenib, a tyrosine kinase inhibitor, possesses potential inhibitory activity against several receptor tyrosine kinases including vascular endothelial growth factor receptor (VEGFR) and platelet-derived growth factor receptor-β (PDGFR-β) [1]. It is an effective chemotherapeutic drug in human hepatocellular, renal, colon and breast cancer [2–5]. However, the efficacy of sorafenib is limited, as it improves only survival rate by three months in hepatocellular carcinoma patients [1, 2]. Furthermore, the anti-cancer effect of sorafenib is inhibited by development of multiple drug resistance mechanisms [6]. Sorafenib resistance was caused by several molecular mechanisms including activation of the epidermal growth factor receptor (EGFR), induction of epithelial-mesenchymal transition (EMT), overexpression of hypoxia inducing factor 1α (HIF1-α) [7–9]. Combination therapy with other less toxic agents could improve the therapeutic efficacy and reduce the drug resistance of sorafenib.

Kahweol, a diterpene molecule from coffee beans, has a variety of bioactivities, including anti-carcinogenesis, anti-tumor, and anti-inflammation properties [10–13]. Anti-carcinogenic properties of kahweol are correlated with the induction of phase II detoxifying and antioxidant enzymes [10]. Our group reported that anti-tumor properties of kahweol may in part be due to inhibition of Akt phosphorylation and activation of JNK signal pathway [14]. Furthermore, kahweol has sensitizing effects of anti-cancer drugs-induced apoptosis. For examples, kahweol sensitizes TRAIL-induced apoptosis through down-regulation of Bcl-2 and c-FLIP expression [15]. Combined treatment with melatonin and kahweol induces apoptosis through up-regulation of PUMA expression [16].

The aim of the present study was to investigate the synergistic effects of kahweol on sorafenib-mediated apoptosis in renal carcinoma Caki cells. The low-dose combination of sorafenib and kahweol enhanced the cytotoxicity by down-regulation of anti-apoptotic protein Mcl-1 and c-FLIP expression.

## RESULTS

### Combined treatment with sorafenib and kahweol induces apoptosis

**Figure 1:**
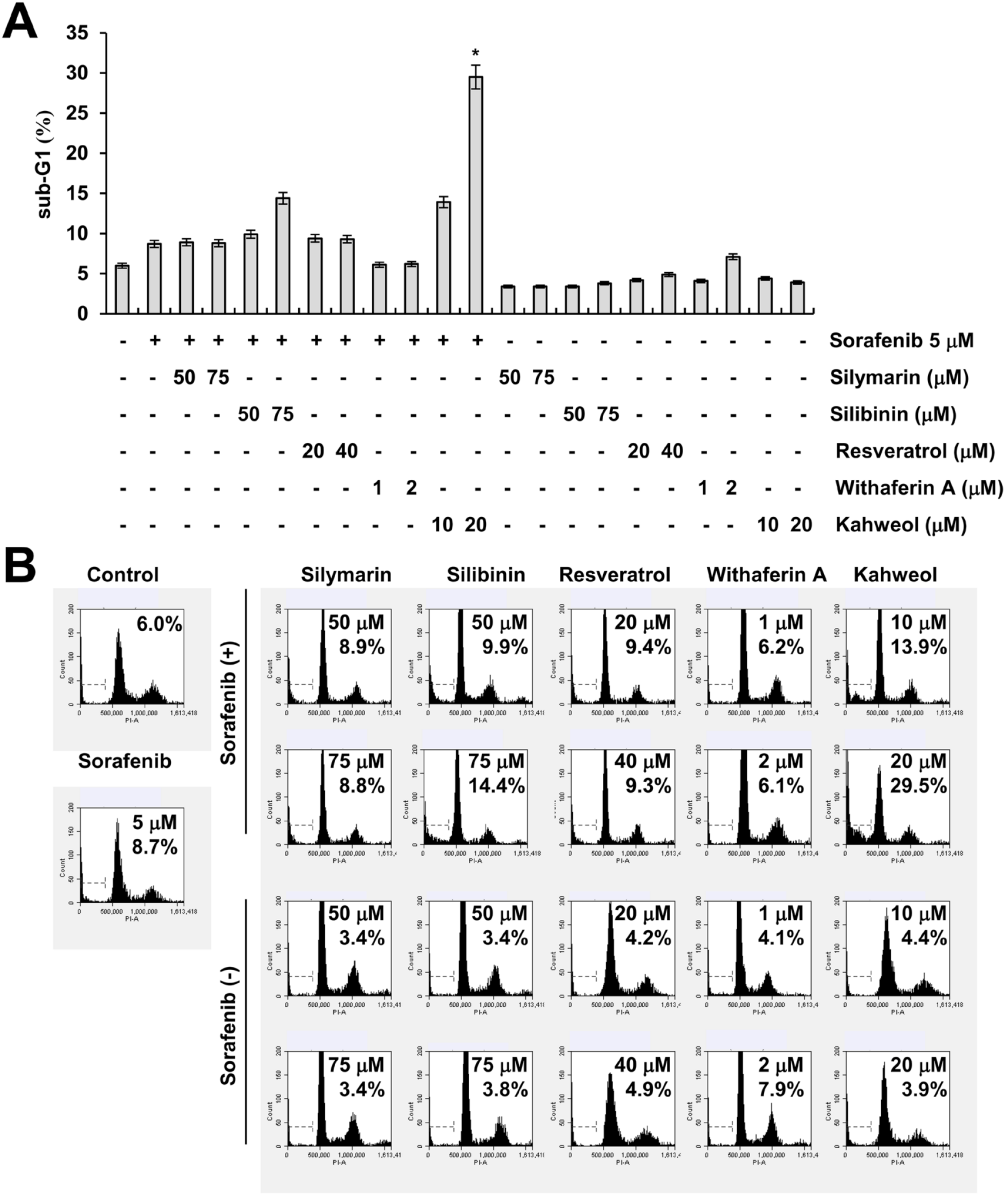
The effect of natural compounds on sorafenib-induced cell death **(A-B)** Caki cells were treated with the indicated concentrations of silymarin, silibinin, resveratrol, withaferin A, and kahweol in the absence or presence of 5 μM sorafenib for 24 h. The sub-G1 fraction was measured by flow cytometry. The values in A represent the mean ± SD from three independent samples.

Sorafenib has anti-cancer effects through inhibiting the RAF-MEK-ERK pathway and receptor tyrosine kinases. However, sorafenib has a many side effects at full dose. Combined sorafenib and other agents reduce dosage of sorafenib, thereby alleviates its side effects. Therefore, we examined whether combined treatment with natural compounds and sorafenib (sub-lethal dosage) induces cell death. As shown in Figure 1A and 1B, among natural compounds, kahweol markedly induced apoptosis dose-dependently in sorafenib-treated cells.

**Figure 2:**
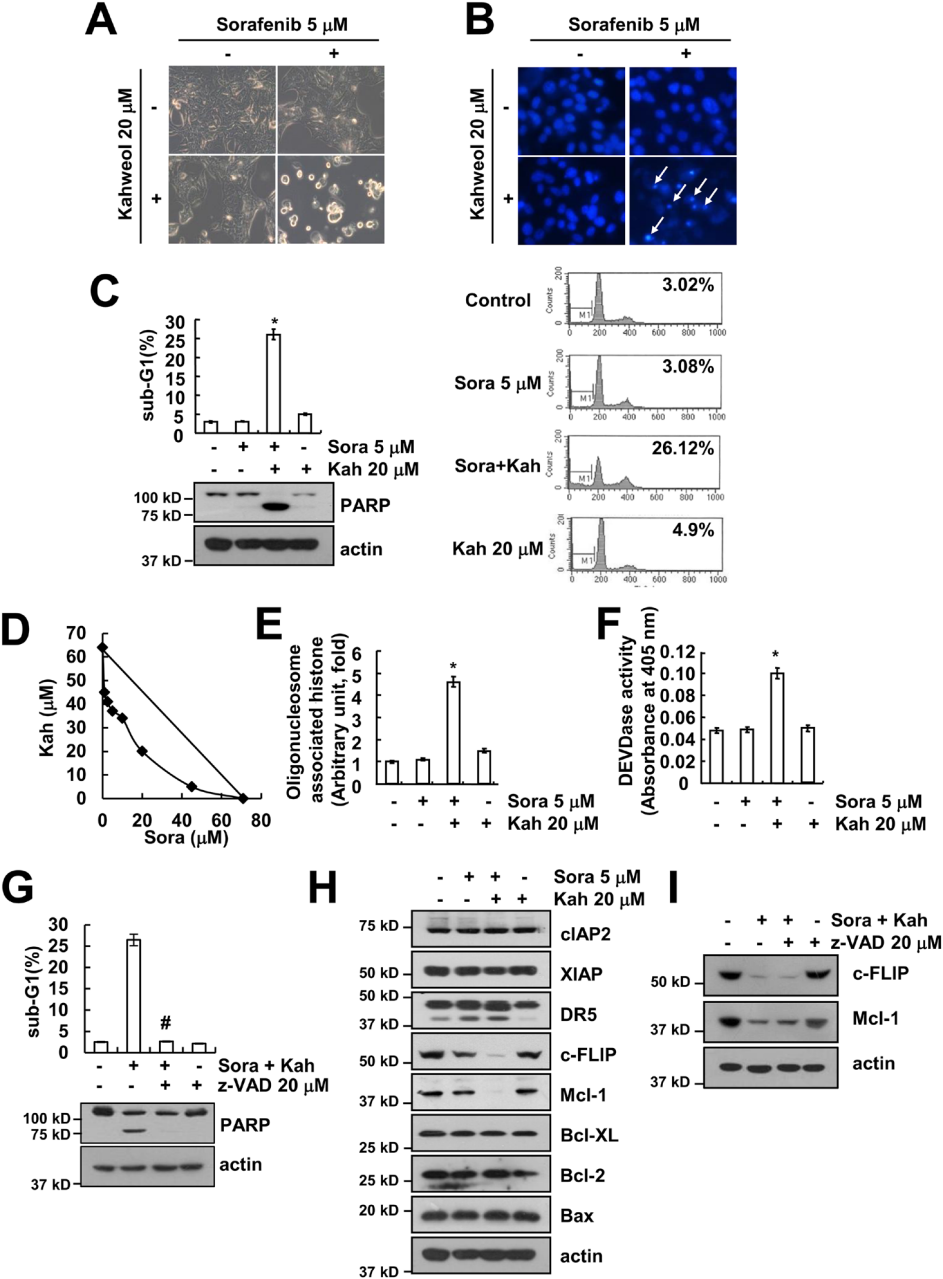
Combined treatment with sorafenib and kahweol induces apoptosis in human renal carcinoma Caki cells **(A-C)** Caki cells were treated with 20 μM kahweol (Kah) in the presence or absence of 5 μM sorafenib (sora) for 24 h. Cell morphology was detected by interference light microscopy **(A)**. The condensation and fragmentation of the nuclei were detected by 4′, 6′-diamidino-2-phenylindole staining **(B)**. The sub-G1 fraction was measured by flow cytometry. The protein expression levels of PARP and actin were determined by Western blot **(C)**. **(D)** Isoboles were obtained by plotting the combined concentrations of each drug required to produce 50% cell death. The straight line connecting the IC_50_ values obtained for the two agents, when applied alone, corresponded to the addition of their independent effects. The values below this line indicate synergy, whereas the values above this line indicate antagonism. **(E-F)** Caki cells were treated with 20 μM kahweol (Kah) in the presence or absence of 5 μM sorafenib (sora) for 24 h. The cytoplasmic histone-associated DNA fragments were determined by a DNA fragmentation detection kit (E). Caspase activities were determined with colorimetric assays using caspase-3 (DEVDase) assay kits (F). **(G)** Caki cells were treated with 20 μM kahweol and 5 μM sorafenib in the presence or absence of 20 μM z-VAD-fmk (z-VAD). The sub-G1 fraction was measured by flow cytometry. The protein expression levels of PARP and actin were determined by Western blotting. **(H)** Caki cells were treated with 20 μM kahweol in the presence or absence of 5 μM sorafenib for 24 h. The protein expression levels of cIAP2, XIAP, DR5, c-FLIP, Mcl-1, Bcl-xL, Bcl-2, Bax, and actin were determined by Western blotting. **(I)** Caki cells were treated with 20 μM kahweol and 5 μM sorafenib in the presence or absence of 20 μM z-VAD-fmk (z-VAD). The protein expression levels of c-FLIP, Mcl-1, and actin were determined by Western blotting. The level of actin was used as a loading control. The values in C, E, F and G represent the mean ± SD from three independent samples. * *p* < 0.01 compared to the control. # *p* < 0.01 compared to the combined treatment with sorafenib and kahweol.

The combination of sorafenib with kahweol induced typical apoptotic morphologies, including blebbing, formation of apoptotic bodies, cell shrinkage, cell detachment on the plate, and chromatin condensation (Figure 2A and 2B). Combined treatment with sorafenib and kahweol markedly induced sub-G1 population and PARP cleavage, which is a substrate of caspase-3 (Figure 2C). Next, we quantified the synergy between the two drugs using Isobologram analysis. Isoboles suggested that sorafenib plus kahweol had synergistic effects (Figure 2D). In addition, combined treatment with sorafenib and kahweol induced cytoplasmic histone-associated DNA fragments (Figure 2E). To further address the caspase activation in kahweol-mediated sensitization to sorafenib-induced apoptosis, we used a pan-caspase inhibitor (z-VAD). Combined treatment markedly increased caspase-3 activation (Figure 2F), and z-VAD blocked sorafenib plus kahweol-induced apoptosis as well as PARP cleavage (Figure 2G). To determine the molecular mechanisms underlying combined treatment-induced apoptosis, we investigated expression levels of apoptosis-related proteins. Both c-FLIP and Mcl-1 expression were down-regulated (Figure 2H). In contrast, other apoptosis-related proteins did not differ in control and combination treated Caki cells. In addition, down-regulation of c-FLIP and Mcl-1 expression was independent of caspase activation (Figure 2I). Collectively, these results indicated that combined treatment with sorafenib and kahweol induces caspase-dependent apoptosis and down-regulation of Mcl-1 and c-FLIP expression.

### Ectopic expression of Mcl-1 overcomes apoptosis in sorafenib plus kahweol-treated cells

**Figure 3:**
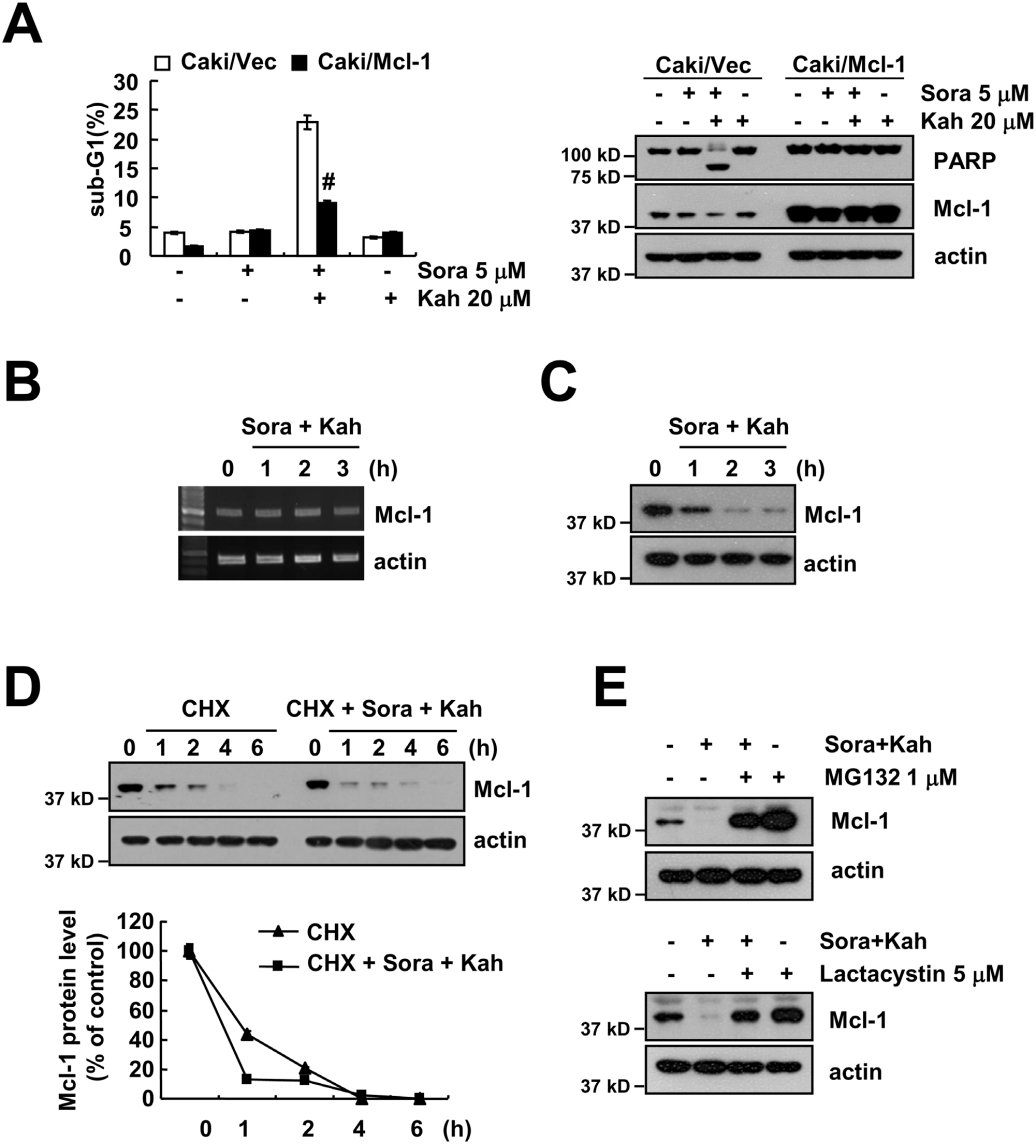
Down-regulation of Mcl-1 expression by sorafenib plus kahweol contributes to apoptosis **(A)** Vector cells (Caki/vector) and Mcl-1-overexpressed cells (Caki/Mcl-1) were treated with 20 μM kahweol (Kah) in the presence or absence of 5 μM sorafenib (sora) for 24 h. The sub-G1 fraction was measured by flow cytometry (left panel). The protein expression levels of PARP, Mcl-1 and actin were determined by Western blot (right panel). **(B-C)** Caki cells were treated with 20 μM kahweol and 5 μM sorafenib for the indicated time periods. The mRNA (B) and protein (C) expression levels of Mcl-1 and actin were determined by RT-PCR and Western blotting, respectively. **(D)** Caki cells were treated with or without 20 μM kahweol and 5 μM sorafenib in the presence of 20 μg/ml cyclohexamide (CHX) for the indicated time periods. The protein expression levels of Mcl-1 and actin were determined by Western blotting. The band intensity of the Mcl-1 protein was measured using ImageJ (public domain JAVA image-processing program ImageJ (http://rsb.info.nih.gov/ij). **(E)** Caki cells were pretreated with 1 μM MG132 and 5 μM lactacystin for 30 min and were then combined with 20 μM kahweol and 5 μM sorafenib for 24 h. The protein expression levels of Mcl-1 and actin were determined by Western blot. The level of actin was used as a loading control. The values in A represent the mean ± SD from three independent samples. # *p* < 0.01 compared to the combined treatment with sorafenib and kahweol-treated Caki/Vec.

To evaluate the significance of Mcl-1downregulation in sorafenib plus kahweol-induced apoptosis, we used Mcl-1 overexpressing cells. When Mcl-1 was overexpressed, the apoptosis population and PARP cleavage caused by sorafenib plus kahweol were markedly inhibited (Figure 3A). Combined treatment with sorafenib and kahweol gradually decreased Mcl-1 expression over 2 h, but the Mcl-1 mRNA expression was not changed (Figure 3B and 3C). Therefore, we investigated whether sorafenib plus kahweol modulates the protein stability of Mcl-1. Caki cells were treated with cycloheximide (CHX) in the presence or absence of sorafenib plus kahweol. As shown in Figure 3D, CHX in the presence of sorafenib plus kahweol rapidly down-regulated expression of Mcl-1 compared with CHX alone. Because Mcl-1 protein stability is well known to be regulated by the ubiquitin-proteasome pathways [17], we examined whether proteasome inhibitors (MG132 and lactacystin) reverse sorafenib plus kahweol-induced Mcl-1 downregulation. Proteasome inhibitors prevented Mcl-1 down-regulation (Figure 3E). These data suggested that the combined treatment induces down-regulation of Mcl-1 expression by the ubiquitin-proteasome pathways and down-regulation of Mcl-1 expression plays a critical role in sorafenib plus kahweol-induced cell death.

### Combined treatment with sorafenib and kahweol induces c-FLIP down-regulation

**Figure 4:**
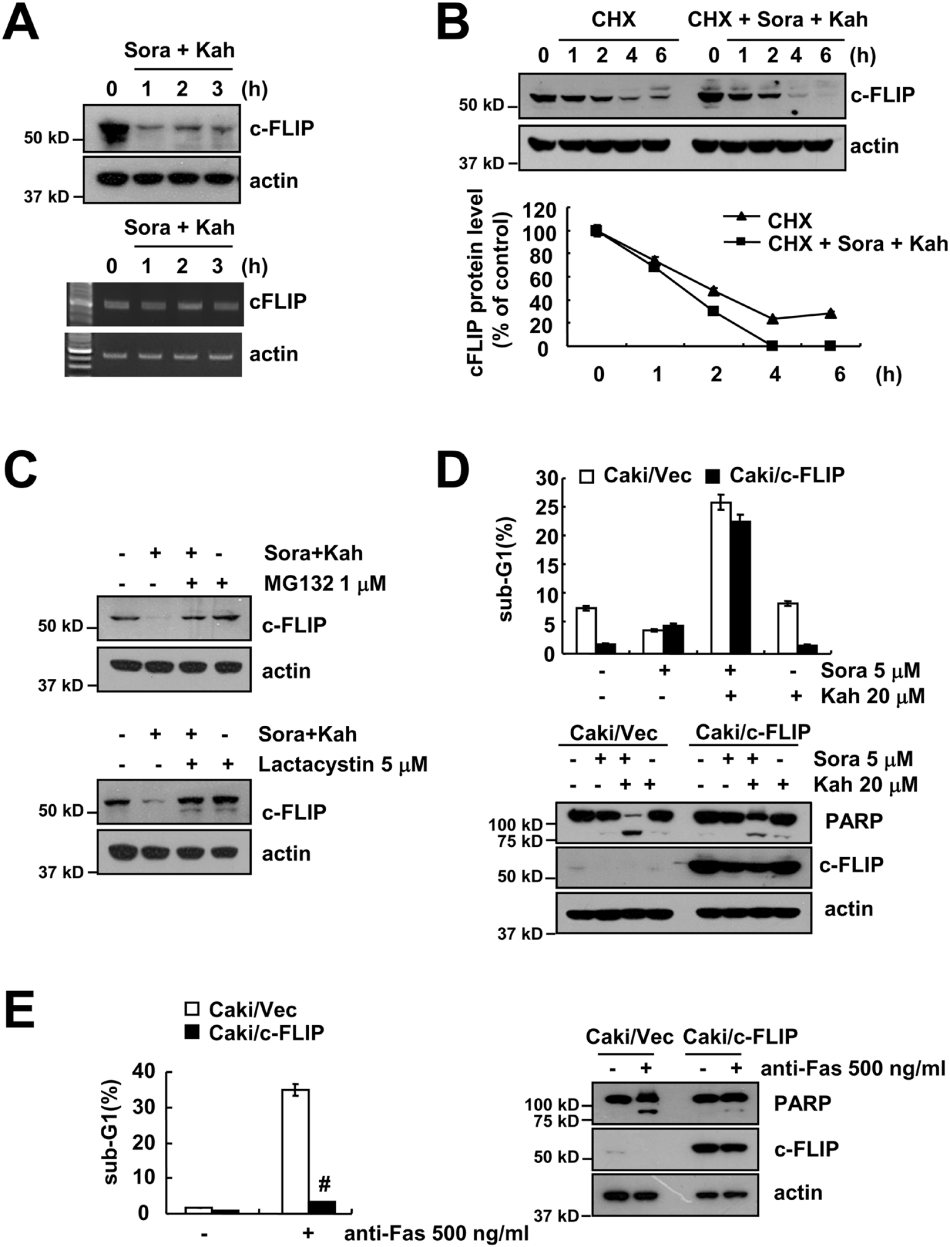
Combined treatment with sorafenib and kahweol overcomes c-FLIP overexpression-mediated resistance **(A)** Caki cells were treated with 20 μM kahweol and 5 μM sorafenib for the indicated time periods. The protein (upper panel) and mRNA (lower panel) expression levels of c-FLIP and actin were determined by Western blotting and RT-PCR, respectively. **(B)** Caki cells were treated with or without 20 μM kahweol and 5 μM sorafenib in the presence of 20 μg/ml cyclohexamide (CHX) for the indicated time periods. The protein expression levels of c-FLIP and actin were determined by Western blotting. The band intensity of the c-FLIP protein was measured using ImageJ (public domain JAVA image-processing program ImageJ (http://rsb.info.nih.gov/ij). **(C)** Caki cells were pretreated with 1 μM MG132 and 5 μM lactacystin for 30 min and were then combined with 20 μM kahweol and 5 μM sorafenib for 24 h. The protein expression levels of c-FLIP and actin were determined by Western blot. **(D)** Vector cells (Caki/vector) and c-FLIP-overexpressed cells (Caki/c-FLIP) were treated with 20 μM kahweol (Kah) in the presence or absence of 5 μM sorafenib (sora) for 24 h. The sub-G1 fraction was measured by flow cytometry. The protein expression levels of PARP, c-FLIP and actin were determined by Western blot. **(E)** Vector cells (Caki/vector) and c-FLIP-overexpressed cells (Caki/c-FLIP) were treated with 500 ng/ml anti-Fas for 24 h. The sub-G1 fraction was measured by flow cytometry. The protein expression levels of PARP, c-FLIP and actin were determined by Western blot. The level of actin was used as a loading control. The values in D and E represent the mean ± SD from three independent samples. # *p* < 0.01 compared to the anti-Fas-treated Caki/Vec.

As shown in Figure 2H, c-FLIP expression was down-regulated by combined treatment. Combined treatment with sorafenib and kahweol did not effect on c-FLIP mRNA expression, but c-FLIP protein levels were rapidly down-regulated after 1 h treatment (Figure 4A). As shown in Figure 4B, CHX alone gradually reduced c-FLIP expression, but combined treatment with CHX and sorafenib plus kahweol more rapidly reduced c-FLIP protein expression. In addition, both proteasome inhibitors also prevented c-FLIP down-regulation (Figure 4C). To evaluate the functional importance of the c-FLIP proteins in sorafenib plus kahweol-induced apoptosis, we used c-FLIP overexpressing cells. In contrast to Mcl-1, overexpression of c-FLIP, surprisingly, did not inhibit sorafenib plus kahweol-induced apoptosis and PARP cleavage (Figure 4D). However, anti-FAS (FAS ligand)-induced apoptosis and PARP cleavage were prevented by ectopic expression of c-FLIP (Figure 4E). Interestingly, these data suggested that combined treatment with sorafenib and kahweol induces apoptotic cell death in c-FLIP overexpressed Caki cells.

### Combined treatment with sorafenib and kahweol increases proteasome activity

**Figure 5:**
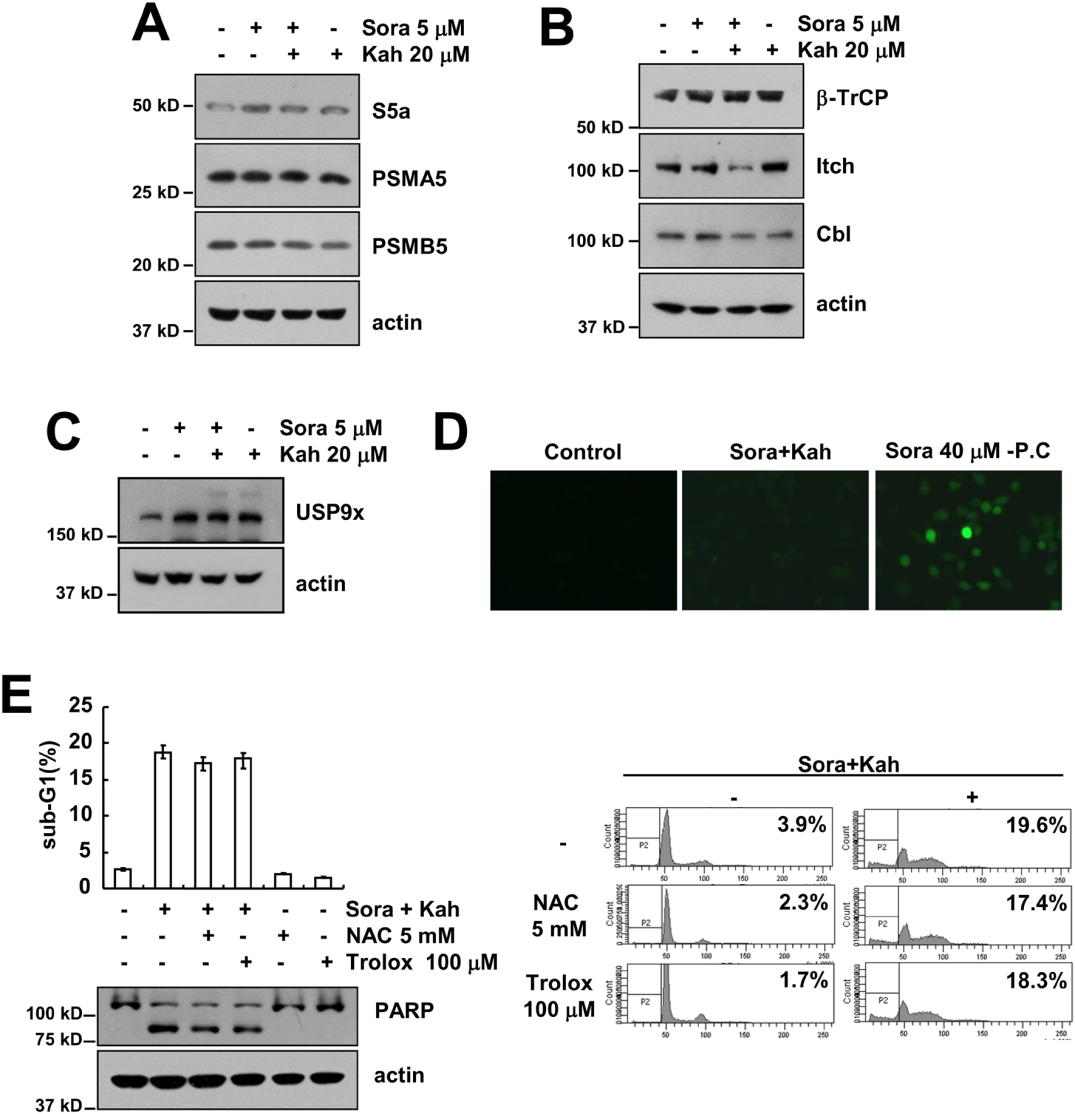
The effects of combined treatment with sorafenib and kahweol on expression of proteasome subunits, E3 ligases, and USP9x and ROS production **(A-C)** Caki cells were treated with 20 μM kahweol in the absence or presence of 5 μM sorafenib for 24 h. The protein expression levels of S5a, PSMA5, PSMB5, and actin were determined by Western blotting (A). The protein expression levels of β-TrCP, Itch, Cbl and actin were determined by Western blotting (B). The protein expression levels of USP9x and actin were determined by Western blotting (C). The level of actin was used as a loading control. **(D)** Caki cells were treated with 20 μM kahweol plus 5 μM sorafenib or 40 μM sorafenib for 3 h. The H_2_DCF-DA fluorescence intensity was detected by a fluorescence microscope. **(E)** Caki cells were pretreated with 5 mM NAC or 100 μM trolox for 30 min and were then treated with 20 μM kahweol plus 5 μM sorafenib for 24 h. The sub-G1 fraction was measured by flow cytometry. The protein expression levels of PARP and actin were determined by Western blotting. The level of actin was used as a loading control. The values in E represent the mean ± SD from three independent samples.

Combined treatment with sorafenib and kahweol reduced Mcl-1 and c-FLIP at the post-transcriptional levels in a proteasome dependent manner (Figures 3E and 4C). Next, we investigated whether combined treatments induced proteasome subunits expression, 19S proteasome non-ATPase regulatory subunit 4 (PSMD4/S5a), and 20S proteasome subunit alpha type 5 (PSMA5) and beta type 5 (PSMB5). However, combined treatment with sorafenib and kahweol did not alter the expression levels of these proteins (Figure 5A). Next, we investigated whether combined treatment specifically modulate E3 ligase of c-FLIP and Mcl-1. However, as shown in Figure 5B, Mcl-1 E3 ligase (β-TrCP) and c-FLIP E3 ligase (Cbl and Itch) was not induced in combined treated cells. Furthermore, deubiquitinase of Mcl-1, USP9x, was not altered by sorafenib and kahweol treatment (Figure 5C). In addition, we investigated whether reactive oxygen species (ROS) is involved in combined treatment-induced apoptosis. As shown in Figure 5D, although high concentrations of sorafenib induced ROS production, combined treatment with sorafenib and kahweol did not induce ROS production. Furthermore, ROS scavengers (NAC and trolox) had no effect on combined treatment-induced apoptosis (Figure 5E). Therefore, combined treatment with sorafenib and kahweol-induced apoptosis is independent of ROS signaling.

### Combined treatment with sorafenib and kahweol induces apoptosis in other cancer cells but not in normal cells

**Figure 6:**
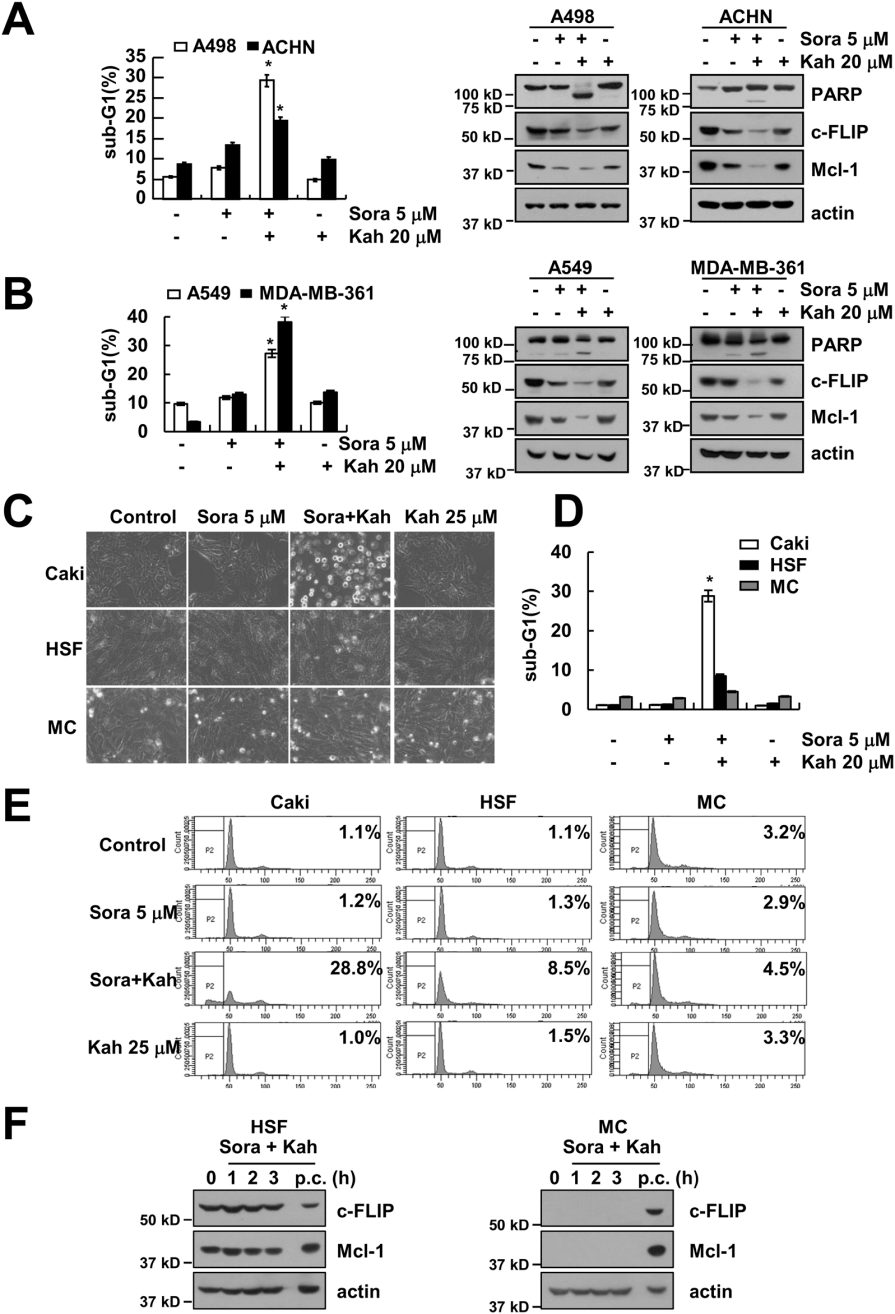
Effect of sorafenib plus kahweol on apoptosis in other cancer cells and in normal cells **(A-D)** Renal carcinoma (A498 and ACHN) (A), lung carcinoma (A549) and breast carcinoma (MDA-MB-361) (B**)** cells were treated with 20 μM kahweol plus 5 μM sorafenib for 24 h. The sub-G1 fraction was measured by flow cytometry. The protein expression levels of PARP, c-FLIP, Mcl-1 and actin were determined by Western blotting. The level of actin was used as a loading control. **(C-E)** Caki cells, human skin fibroblast (HSF), and normal mesangial cells (MC) were treated with 20 μM kahweol plus 5 μM sorafenib for 24 h. The cell morphology was examined using interference light microscopy (C). The sub-G1 fraction was measured by flow cytometry (D-E**)**. **(F)** HSF and MC cells were treated with 20 μM kahweol plus 5 μM sorafenib for the indicated time periods. (p.c : positive control; Caki cell lysates). The protein expression levels of c-FLIP, Mcl-1 and actin were determined by Western blotting. The values in A, B and D represent the mean ± SD from three independent samples. * *p* < 0.01 compared to the control.

We next investigated the effect of sorafenib plus kahweol on apoptosis in other renal cancer cells (A498 and ACHN cells) and other type cancer cells, including human lung carcinoma (A549) and breast carcinoma (MDA-MB-361) cells. We found that combined treatment with sorafenib and kahweol induced apoptotic cell death and cleavage of PARP in A498, ACHN, A549, and MDA-MB-361 cells (Figure 6A and 6B). Furthermore, sorafenib plus kahweol induced down-regulation of Mcl-1 and c-FLIP expression (Figure 6A and 6B). By contrast, combined treatment with sorafenib and kahweol did not induce morphological changes, apoptosis, and expression of Mcl-1 and c-FLIP expression in normal human mesangial cells (MC) and normal human skin fibroblast cells (HSF) (Figure 6C–6F).

## DISCUSSION

Combination of natural compounds and conventional anti-cancer drug at their lower individual concentrations markedly enhances efficacy in induction of apoptotic cell death. Recent studies reported that sorafenib combination treatment with resveratrol, indol-3-carbinol, bufalin, fisetin, and triptolide effectively induced apoptosis in various carcinoma cells by different mechanisms [18–22]. In this study, the combined treatment with low dose sorafenib and kahweol induced apoptotic cell death in cancer cells, but not in normal cells. Combined treatment with sorafenib and kahweol potentiated the cytotoxicity in renal carcinoma cells through down-regulation of Mcl-1 and c-FLIP protein expression in a proteasome-dependent manner. Our study is the first to show the combined treatment with sorafenib and kahweol may be effective in overcoming resistance to anti-cancer drugs in renal carcinoma cells.

Numerous investigations have been reported that generation of intracellular ROS plays a critical a role in apoptosis [23]. Interestingly, a high concentration of kahweol (75 μM) induced ROS production in breast carcinoma MDA-MB-231 cells [24]. However, a low concentration of kahweol (25 μM) did not increase ROS production in our experiment. In addition, as shown in Figure 5E, pretreatment with NAC or trolox did not prevent combined treatment-induced sub-G1 population and cleavage of PARP. Therefore, ROS is not critical for the induction of apoptosis by combined treatment with sorafenib and kahweol.

Mcl-1 and c-FLIP protein expression are regulated at the transcriptional and post-translational levels [17, 25–27]. Combined treatment with sorafenib and kahweol did not alter Mcl-1 and a c-FLIP mRNA level, suggesting that down-regulation of Mcl-1 and c-FLIP protein is likely to be a post-transcriptional event. Interestingly, inhibition of proteasome with MG132 or lactacystin rescued Mcl-1 and c-FLIP down-regulation (Figures 3E and 4C). Combined treatment with sorafenib and kahweol induced down-regulation of c-FLIP and Mcl-1 expression by proteasomal degradation (Figures 3E and 4C), but combined treatment with sorafenib and kahweol did not increase proteasome activity (Data not shown; negative data). Furthermore, combined treatment did not change expression levels of subunits of proteasome catalytic core proteins (Figure 5A). A key player in ubiquitin-mediated protein degradation is the E3 ubiquitin ligase [28]. To date, four E3 ligases [Mcl-1 Ubiquitin ligase E3 (MULE), beta transducin-containing protein (β-TrCP), F-box and WD repeat domain-containing 7 (FBW7), and Tripartite motif containing 17 (Trim17)] and one deubiquitinase (USP9x) have been involved in ubiquitin-mediated Mcl-1 regulation [17, 29–32]. The E3 ligase Casitas B-lineage lymphoma (Cbl) and Itch have been implicated in c-FLIP stability control [25, 33]. We also investigated the E3 ligase expression of c-FLIP and Mcl-1. However, combined treatment with sorafenib and kahweol did not change E3 ligase expression of β-TrCP, Itch and Cbl (Figure 5B). How other E3 ligase or unknown E3 ligase in post-translational regulation of c-FLIP and Mcl-1 protein affect apoptotic cell death may need further investigation.

Collectively, these results suggest that sorafenib plus kahweol induced apoptosis through down-regulating Mcl-1 expression at post-translational levels. Therefore, it uncovered a critical role of kahweol in enhancing the anti-cancer effect of sorafenib, suggesting that combined treatment with sorafenib and kahweol offers a rationale for a new therapeutic combination for the treatment of renal carcinoma.

## MATERIALS AND METHODS

### Cell culture and materials

Human renal carcinoma (Caki, ACHN and A498), human lung carcinoma (A549), and human breast carcinoma (MDA-MB-361) cells were obtained from the American Type Culture Collection (Manassas, VA, USA). Primary cultured human mesangial cells (MC) (Cryo NHMC) were purchased from Clonetics (San Diego, CA). The normal human skin fibroblasts (HSFs) cells were purchased form Korea Cell Line Bank (Seoul, Korea). The culture medium used throughout these experiments was Dulbecco's modified Eagle's medium (DMEM) or RPMI containing 10% fetal bovine serum (FBS), 20 mM HEPES buffer and 100 μg/mL gentamycin. The PCR primers were purchased from Macrogen (Seoul, Korea). The z-VAD-fmk was purchased from R&D system (MN, USA), and N-acetyl-L-cysteine (NAC) and Trolox was obtained from Calbiochem (San Diego, CA, USA). Sorafenib was purchased from selleck chemicals (Houston, TX, USA). Resveratrol and withafectin A were obtained from Biomol International (Plymouth Meeting, PA, USA). Kahweol was purchased from LKT (St. Paul, MN, USA). Anti-Bax and anti-XIAP antibodies were purchased from BD Biosciences (Bedford, MA). Anti-Bcl-2, anti-Cbl, anti-Bcl-xL, anti-Mcl-1, anti-cIAP2, anti-β-TrCP, and anti-Itch antibodies were purchased from Santa Cruz Biotechnology (CA, USA). Anti-DR5, anti-PARP, anti-PSMA5, anti-PSMB5, and anti-S5A were obtained from Cell Signaling Technology (Beverly, MA, USA). The Fas agonist monoclonal antibody was purchased from upstate biotechnology (Lake Placid, NY, USA). Anti-c-FLIP antibody was obtained from ALEXIS Corporation (San Diego, CA). Anti-actin antibody and other chemicals were obtained from Sigma Chemical Co. (St. Louis, MO, USA). Human Mcl-1 and c-FLIP expression vectors were constructed as described previously [34, 35].

### Flow cytometry analysis

For flow cytometry, the cells were resuspended in 100 μl of phosphate-buffered saline (PBS), and 200 μl of 95% ethanol was added while the cells were being vortexed. Then, the cells were incubated at 4°C for 1 h, washed with PBS, resuspended in 250 μl of 1.12% sodium citrate buffer (pH 8.4) with 12.5 μg of RNase and incubated for an additional 30 min at 37°C. The cellular DNA was then stained by adding 250 μl of a propidium iodide solution (50 μg/ml) to the cells for 30 min at room temperature. The stained cells were analyzed by fluorescent-activated cell sorting on a FACScan flow cytometer to determine the relative DNA content, which was based on the red fluorescence intensity.

### 4′,6′-Diamidino-2-phenylindole staining (DAPI) for nuclei condensation and fragmentation

To examine the cellular nuclei, the cells were fixed with 1% paraformaldehyde on glass slides for 30 min at room temperature. After fixation, the cells were washed with PBS and a 300 nM 4′,6′-diamidino-2-phenylindole solution (Roche, Mannheim, Germany) was added to the fixed cells for 5 min. After the nuclei were stained, the cells were examined by fluorescence microscopy.

### Western blot analysis

For the Western blot experiments, the cells were washed with cold PBS and lysed on ice in modified RIPA buffer (50 mM Tris-HCl pH 7.4, 1% NP-40, 0.25% Na-deoxycholate, 150 mM NaCl, 1 mM Na_3_VO_4_, and 1 mM NaF) containing protease inhibitors (100 μM phenylmethylsulfonyl fluoride, 10 μg/ml leupeptin, 10 μg/ml pepstatin, and 2 mM EDTA). The lysates were centrifuged at 10,000 x *g* for 10 min at 4°C, and the supernatant fractions were collected. The proteins were separated by SDS-PAGE electrophoresis and transferred to Immobilon-P membranes. The specific proteins were detected using an enhanced chemiluminescence (ECL) Western blotting kit according to the manufacturer’s instructions.

### Determination of synergy

The possible synergistic effect of sorafenib and kahweol was evaluated using the isobologram method. In brief, cells were treated with different concentrations of sorafenib and kahweol alone or in combination. After 24 h, relative survival was assessed, and the concentration effect curves were used to determine the IC50 (the half-maximal inhibitory concentration) values for each drug alone and in combination with a fixed concentration of the second agent. The XTT assay was employed to measure cell viability using a WelCount Cell Viability Assay Kit (WelGENE, Daegu, Korea). In brief, the reagent was added to each well and was then measured with a multi-well plate reader (at 450 nm/690 nm).

### The DNA fragmentation assay

A cell death detection ELISA plus kit (Boehringer Mannheim; Indianapolis, IN) was used to determine the level of apoptosis by detecting fragmented DNA within the nuclei of kahweol-treated cells, sorafenib-treated cells, or cells that were treated with a combination of sorafenib and kahweol. Briefly, each culture plate was centrifuged for 10 min at 200 × *g*, the supernatant was removed, and the cell pellet was lysed for 30 min. Then, the plate was centrifuged again at 200 × *g* for 10 min and the supernatant, which contained the cytoplasmic histone-associated DNA fragments, was collected and incubated with an immobilized anti-histone antibody. The reaction products were incubated with a peroxidase substrate for 5 min and were measured by spectrophotometry at 405 and 490 nm (reference wavelength) with a microplate reader. The signals in the wells containing the substrate alone were subtracted as the background.

### Asp-Glu-Val-Asp-ase (DEVDase) activity assay

To evaluate the DEVDase activity, cell lysates were prepared after their respective treatments with sorafenib in the presence or absence of kahweol. Assays were performed in 96-well microtiter plates by incubating 20 μg of the cell lysates in 100 μl of reaction buffer (1% NP-40, 20 mM Tris-HCl, pH 7.5, 137 mM NaCl, 10% glycerol) containing a caspase substrate [Asp-Glu-Val-Asp-chromophore-p-nitroanilide (DVAD-pNA)] at 5 μM. The lysates were incubated at 37°C for 2 h. Thereafter, the absorbance at 405 nm was measured with a spectrophotometer.

### Reverse transcription polymerase chain reaction (RT-PCR)

Total RNA was isolated using the TriZol reagent (Life Technologies; Gaithersburg, MD), and the cDNA was prepared using M-MLV reverse transcriptase (Gibco-BRL; Gaithersburg, MD) according to the manufacturers’ instructions [36, 37]. The following primers were used for the amplification of human c-FLIP, Mcl-1 and actin: c-FLIP (sense) 5′- CGG ACT ATA GAG TGC TGA TGG -3′ and (antisense) 5′- GAT TAT CAG GCA GAT TCC TAG -3′; Mcl-1 (sense) 5′- GCG ACT GGC AAA GCT TGG CCT CAA-3′ and (antisense) 5′- GTT ACA GCT TGG ATC CCA ACT GCA-3′; and actin (sense) 5′- GGC ATC GTC ACC AAC TGG GAC -3′ and (anti-sense) 5′- CGA TTT CCC GCT CGG CCG TGG -3′. PCR amplification was carried out using the following cycling conditions: 94°C for 3 min followed by 17 (actin) or 23 cycles (c-FLIP and Mcl-1) of 94°C for 45 s; 58°C for 45 s; 72°C for 1 min; and a final extension at 72°C for 10 min. The amplified products were separated by electrophoresis on a 1.5% agarose gel and detected under UV light.

### Measurement of reactive oxygen species (ROS)

Intracellular accumulation of ROS was determined using the fluorescent probes 2′, 7′-dichlorodihydrofluorescein diacetate (H_2_DCFDA) and Mitosox Red. The Caki cells were treated with sorafenib plus kahweol, and then, the cells were stained with the H_2_DCFDA fluorescent dye for an additional 10 min, followed by trypsinization and resuspension in PBS. The fluorescence was measured at specific time intervals with fluorescence microscope (Zeiss, NY, USA).

### Statistical analysis

The data were analyzed using one-way ANOVA and post-hoc comparisons (Student-Newman-Keuls) using the Statistical Package for Social Sciences 22.0 software (SPSS Inc.; Chicago, IL, USA).

## Abbreviations

CHX: cycloheximide
PSMA5: 20S proteasome subunit alpha type 5
ROS: reactive oxygen species
NAC: N-acetylcysteine
